# Patient Satisfaction, Recommendation Rate, and Patient Comfort With an FDA-Cleared Cryolipolysis System

**DOI:** 10.1093/asjof/ojac067

**Published:** 2022-08-12

**Authors:** Jens Altmann, Felix Jehle, Werner Mang

**Affiliations:** In private practice, Lindau, Germany; In private practice, Lindau, Germany; In private practice, Lindau, Germany

## Abstract

**Background:**

Cryolipolysis is a non-surgical procedure for subcutaneous fat layer reduction by controlled cooling. During the past few years, the use of cryolipolysis for non-invasive body contouring has increased significantly.

**Objectives:**

This retrospective study examines patient satisfaction, recommendation rate and patient comfort with the use of an FDA-cleared system (CoolSculpting Elite, Allergan Aesthetics, AbbVie Company, Irvine CA) for cryolipolysis at a single clinic and reports on the results.

**Methods:**

Between December 2020 and January 2022, 91 patients were treated with an FDA-cleared cryolipolysis system. To assess patient satisfaction, patients were asked to complete clinical questionnaires three months after their last treatment session. The following questions were asked: painfulness of the treatment, complications, satisfaction with the treatment, consideration of further treatment sessions, and willingness to further recommend the treatment.

**Results:**

Eighty-four percent of the 91 patients were female, and 16% were male. The average age was 45.5 years and the mean BMI was 26 kg/m^2^. Patients rated the treatment in terms of pain and discomfort experienced during the procedures on a scale of 1 to 5, with the value 1 representing not painful and 5 as extremely painful. 40% of the patients evaluated the procedure with 1, 38% with 2, 19% with 3, 1% with 4 and 2% with 5. With respect to satisfaction rates, 66% rated the treatment on a scale from 1 to 5 with 1, 18% with 2, 7% with 3, 7% with 4 and 3% with 5, with 1 indicating very satisfied and 5 indicating very dissatisfied. Thus, the overall level of satisfaction (the sum of scale values 1 and 2) amounts to 84%. Of 91 patients, 88% would agree to further treatment and 92% would recommend the therapy to others. All patients reported temporary tissue reactions such as swelling and redness which did not require any further treatment and were self-limiting. Serious or permanent complications did not occur.

**Conclusions:**

The results of our study show that cryolipolysis is a safe and effective method for non-surgical body contouring, providing a high degree of patient satisfaction and recommendation rate.

**Level of Evidence: 4:**

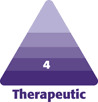

Today there is an increasing demand for non-surgical treatment methods for body contouring, both for women and men. In recent years, the outlined interest in aesthetic treatments corresponds with the increase in non-invasive therapies. According to the International Association for Aesthetic-Plastic Surgery (ISAPS) 13,618,735 non-surgical and 11,363,569 surgical procedures were performed worldwide in 2019. Compared to the 2018 survey, there was a 7,6% increase in non-surgical treatments.^1^ This trend was confirmed by a survey carried out by the American Association for Dermatologic Surgery (ASDS) in 2019, in which almost 70% of respondents said that they were considering a medical-aesthetic treatment. The main reason patients desired treatment was shedding disturbing extra weight on the body (84%) or the submental region (73%).^2^ The results of both surveys are consistent with the increasing demand for body contouring through non-surgical treatments. According to surveys by the American Society for Dermatologic Surgery, 257,868 cryolipolysis treatments were carried out in 2019. This figure represents an increase of 214% since 2012.^2^ According to “The Aesthetic Society”, cryolipolysis was the seventh most common non-surgical aesthetic procedure in 2021.^3^

Historically, the name “cryolipolysis” is based on the clinical observations of cold-induced panniculitis.^4-6^ These observations led to the assumption that fat cells are “more susceptible to cold injury” than other cells. As a result, Manstein et al. developed a non-invasive treatment for subcutaneous fat deposits in 2007 and named it “selective cryolysis."^7^ Previous studies showed that cryolipolysis is a safe and effective non-invasive method for “subcutaneous fat reduction."^8-15^ The most frequently cited reasons by patients who chose cryolipolysis treatment at our clinic were: no anaesthesia required, no subsequent surgical scarring, minimal recovery time, no hospitalization and fewer psychological barriers.

## METHODS

This study reports the results of a retrospective survey with regard to patient satisfaction, recommendation rate and patient comfort with treatment using an FDA-cleared cryolipolysis system (CoolSculpting Elite, Allergan Aesthetics AbbVie Company, Irvine, CA) at a single clinic. Between December 2020 and January 2022, 91 consecutive patients were treated. In order to evaluate patient satisfaction, patients were surveyed by clinic questionnaires three months after their last therapy session, either within the course of a follow-up appointment or in a telephone interview. The study protocol followed both the Declaration of Helsinki 1975 and the national legal regulations (Art. 27 Abs. 4 BayKrG).

The following questions were asked: painfulness of the treatment, complications, treatment satisfaction, consideration of further therapy, and treatment recommendation. The pain level was assessed with a scale from 1 to 5, whereby 1 represented not painful and 5 extremely painful. Satisfaction with the treatment was surveyed on a scale of 1-5, whereby 1 corresponded to very satisfied, 2 satisfied, 3 neutral, 4 unsatisfied and 5 very unsatisfied. Furthermore, the questionnaires were allocated according to the following patient criteria: male or female, age, ethnic origin, body-mass-index, as well as the number and distribution of treatment cycles.

The standard procedure for treatment with cryolipolysis in our clinic is structured as follows: the patient is invited to attend an initial medical consultation, during which a detailed medical history is requested and a physical examination is carried out, taking into account inclusion and exclusion criteria. The patient discusses his wishes and expectations to match his own expectations with the medically possible results. During the whole-body assessment all the affected body areas are identified with regard to applicators, the number of treatment cycles and treatments. The treatment is carried out by medical personnel under supervision of a physician (Video). The treatment area is examined and the applicator positions are marked.

At the end of each treatment, the treated area is massaged for 2 min. Subsequently, the patient is instructed to continue the massage of the treated area twice a day for the following 14 days. Three minutes after the start of the first session, at the end of the cycle and after the massage, the patient is asked about physical comfort and the degree of pain/discomfort. If the patient generally feels well, he or she is discharged from cryolipolysis treatment. After 3 months, a follow-up examination with photo documentation and evaluation by the clinic’s internal survey questionnaire follows. Should the patient be unable to attend the check-up in person, it can also be done by telephone.

## RESULTS

During the period of the retrospective study 91 patients were treated. The standardized follow-up time was 3 months after the last treatment session. Eighty-four percent (*n* = 76) of the study population were female and 16% were male (*n* = 15).

The average age of both women and men was 45.5 years. The youngest female patient was 21 years old and the oldest was 67 years old. Among the male patients, the youngest was 24 years old and the oldest was 57 years old.

**Figure 1.**
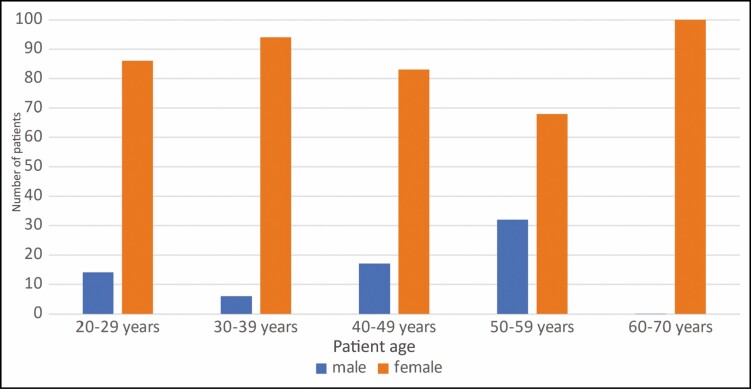
Patient cohort stratified according to age and gender groups (in %).

In the 20-29 years age group, there were 12 patients, of whom 83% were female and 17% male. In the age group between 30 and 39 years, 93% were female and 7% male. The age group 40-49 years contained the most patients, numbering 28. Among these, 82% were female and 18% were male. In the age group 50-59 years, there was a total of 23 patients, of whom 68% were female and 32% were male. The age group 60-69 years contained 13 patients, all of whom were female (100%) (Figure 1). The ethnic distribution of the patient cohort in our study was 100% Caucasian.

The average BMI was 26 kg/m^2^. Among our female patients, the average was 25 kg/m^2^ and in male patients, the average was 28 kg/m^2^. The mean weight before the treatment was 72.9 kg and after 72.8 kg. The difference in weight in our patients was found to be not statistically significant to 0 in a paired two-sided *t*-test (*P* = 0.3636).

From December 2020 until January 2022 (*n* = 13 months), 91 patients participating in the study were treated with 307 treatment cycles on various areas of the body. Most of the treatment cycles (*n* = 131) pertained to the abdomen (43%). The further treatment cycles were distributed among the flanks (11%, *n* = 34), inner thighs (9%, *n* = 28), outer thighs (8%, *n* = 24), banana rolls (7%, *n* = 21) upper arms (6%, *n* = 20), inner knee (6%, *n* = 18), submental area (5%, *n* = 14), breast (3%, *n* = 8), front thighs (2%, *n* = 7), and pre-axillary area (1%, *n* = 2).

**Figure 2.**
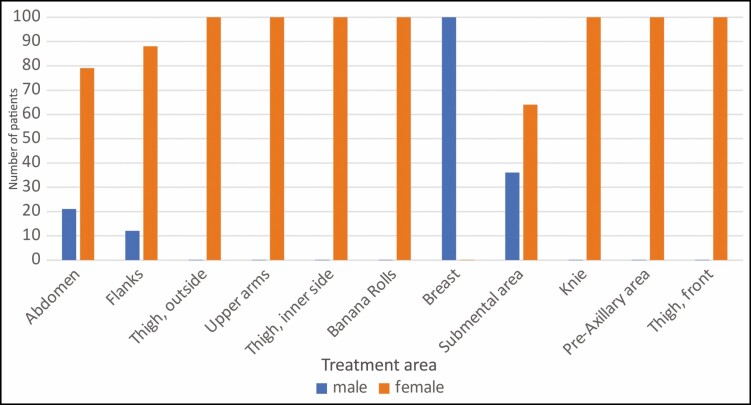
Gender distribution of treatment areas.

The gender distribution of the treated body sites (Figure 2) shows that male patients focused on treatments of the abdomen (21%), flanks (12%), breast (100%), and submental area (36%). Treatment of extremities such as thighs and knees, banana rolls, and upper arms was100% requested by women. The further body area distribution among females related to treatment cycles for the abdomen (79%) and flanks (88%).

### Patient Safety and Comfort

To evaluate patient safety and patient comfort, patients were again asked to report any side effects 3 months after the end of treatment in a questionnaire administered by our clinic. Furthermore, patient comfort was documented by assessing the degree of pain during treatment. The pain scale of 1-5 was evaluated and documented, whereby 1 represented not painful and 5 represented extremely painful.

Patients reported temporary side effects such as redness and pain/discomfort both throughout the study, as well as in the follow-up period, but these, however, completely subsided within 2 weeks after the end of the treatment. Severe or permanent complications did not occur. An emergency telephone number is always available for all clinic patients, regardless of whether the treatment is non-invasive or invasive.

**Figure 3.**
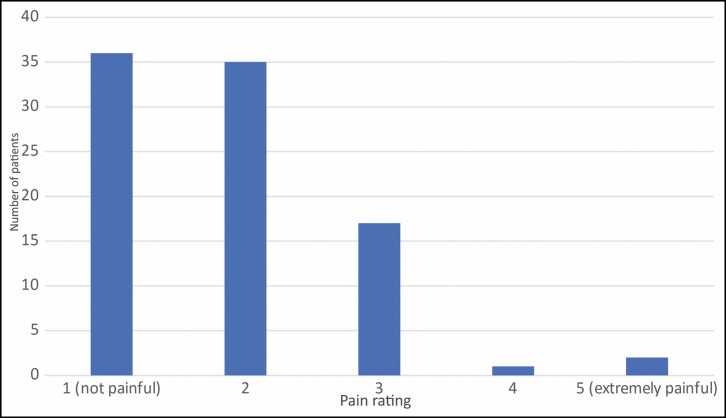
Subjective painfulness of treatment.

The degree of pain was evaluated by 40% of patients with 1, 38% with 2, 19% with 3, 1% with 4 and 2% with 5 (Figure 3). There was no case of discontinuation of treatment due to the level of pain or patient discomfort.

### Patient Satisfaction and Recommendation Willingness

**Figure 4.**
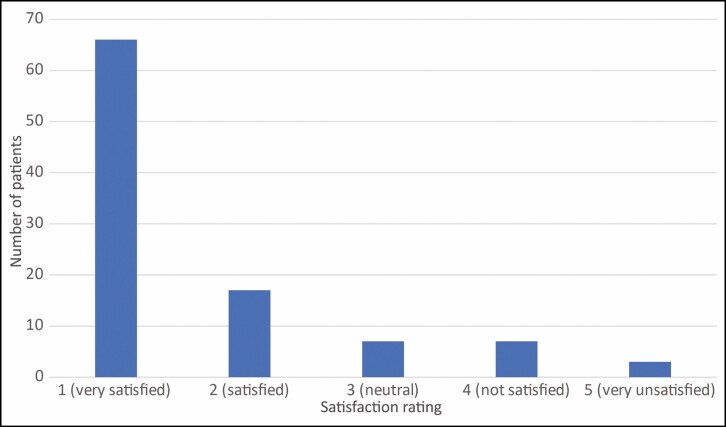
Patient satisfaction.

**Figure 5.**
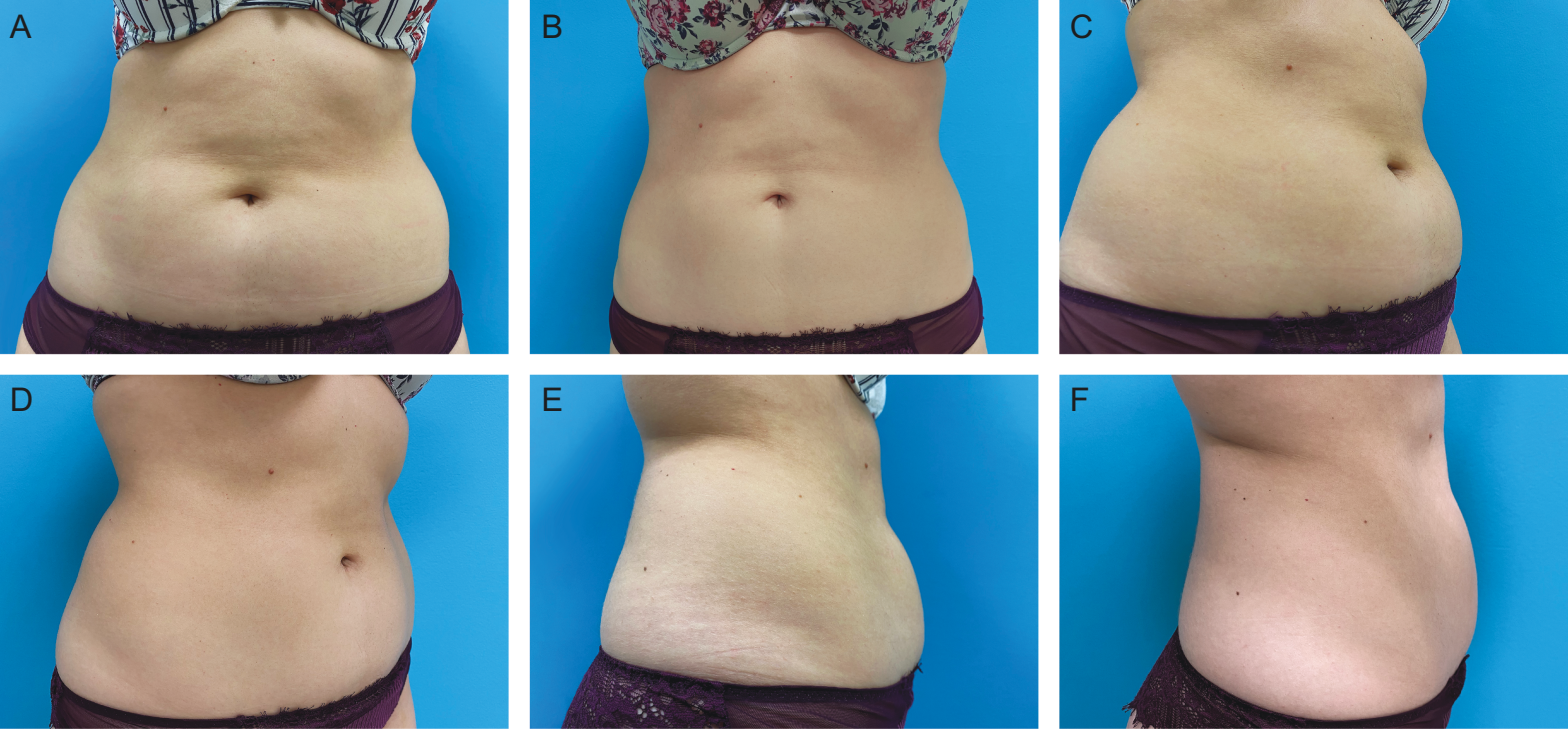
Abdominal cryolipolysis treatment shown on a 28-year-old female patient; weight before and after the procedure was 58 kg. (A, C, E) Pretreatment and (B, D, F) 3 months after cryolipolysis treatment of the lower abdomen and flank shown from anterior, oblique, and lateral views, respectively. Applicators: lower abdomen 2 C150, flanks 2 C120.

**Figure 6.**
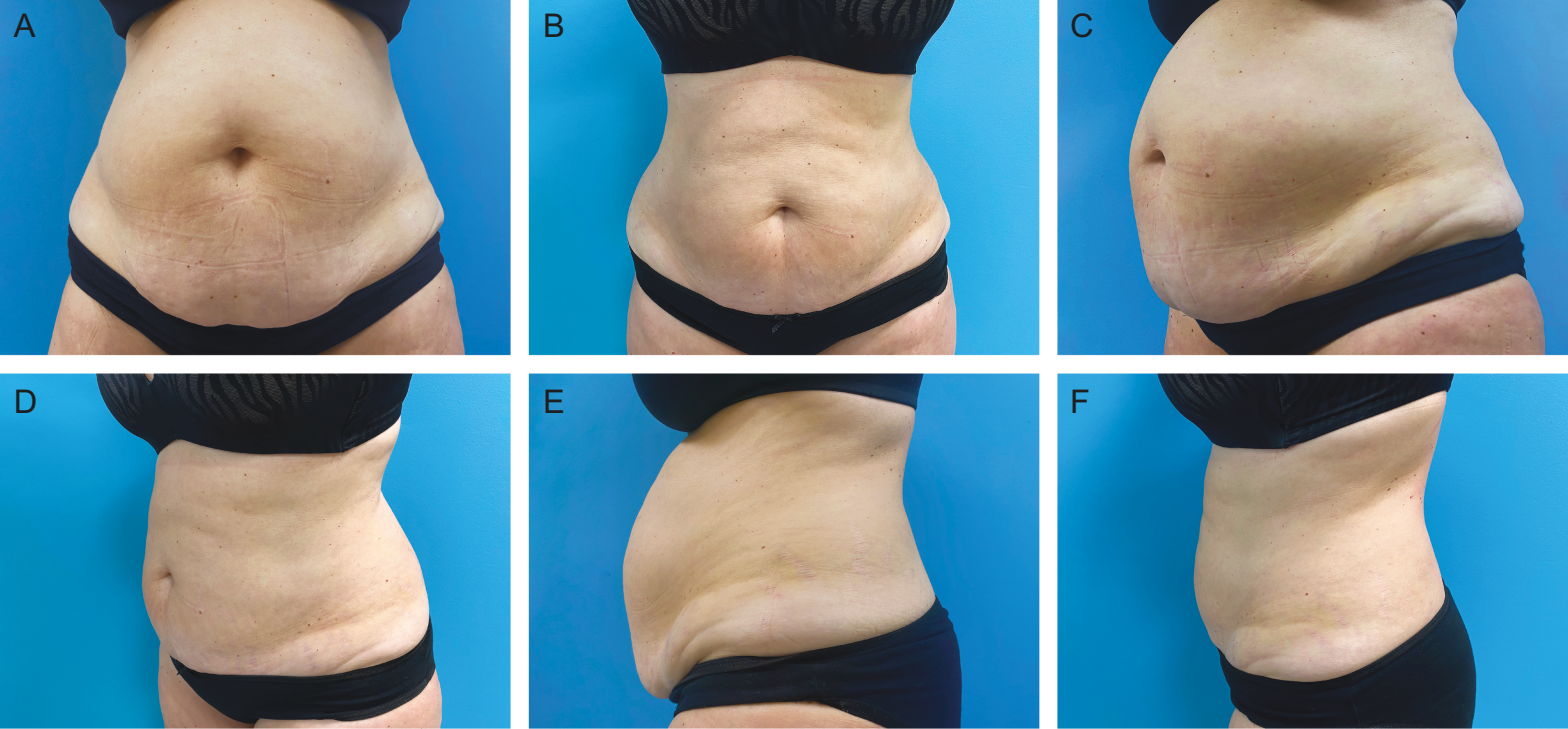
Abdominal cryolipolysis treatment shown on a 56-year-old female patient; weight before and after the procedure was 70 kg. (A, C, E) Pretreatment and (B, D, F) 3 months after cryolipolysis treatment of the abdomen shown from anterior, oblique, and lateral views, respectively. Applicators: upper abdomen 2 C240, lower central abdomen 1 C240.

**Figure 7.**
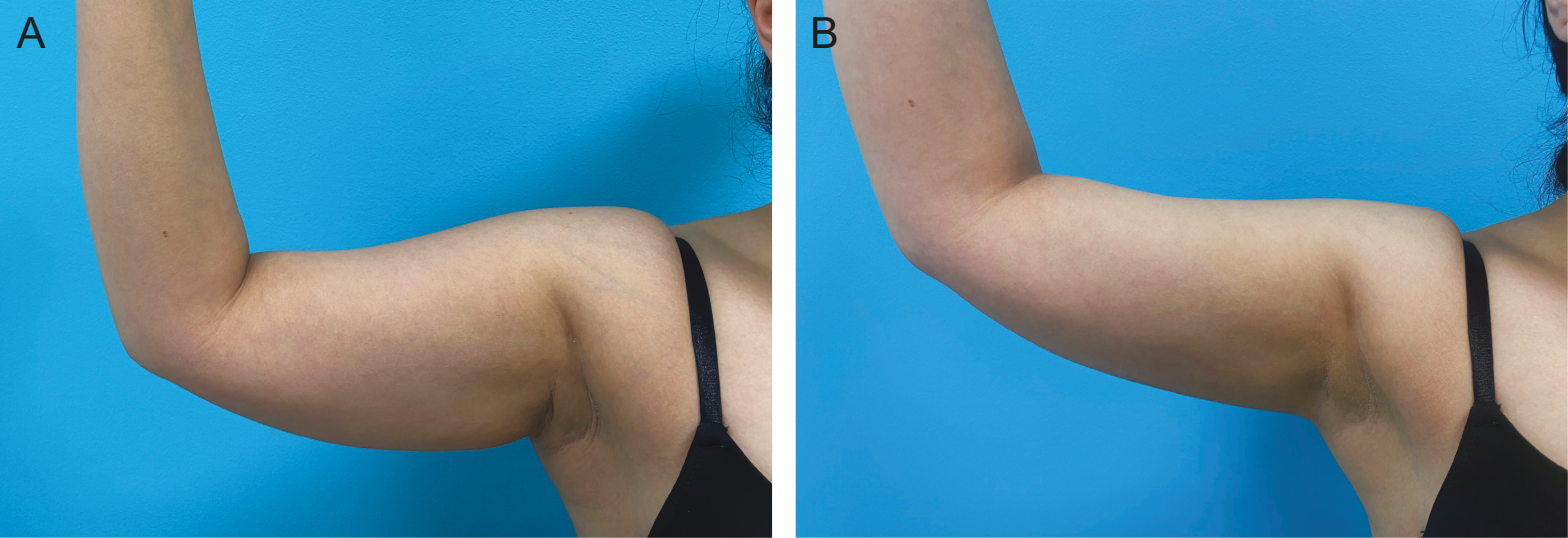
Upper arm cryolipolysis treatment shown on a 31-year-old female patient; weight before and after the procedure was 60 kg. (A) Pretreatment and (B) 3 months after cryolipolysis treatment of the upper arms. Applicators: upper arms 2 F125 per arm.

Patient satisfaction, recommendation rate as well as the personal willingness for continuing the treatment were surveyed by the questionnaire three months after the end of treatment. Satisfaction with the treatment was surveyed on a scale of 1-5, whereby 1 corresponded to very satisfied, 2 satisfied, 3 neutral, 4 unsatisfied, and 5 very unsatisfied. 66% of patients (*n* = 60) evaluated their satisfaction with 1, 18% (*n* = 16) with 2, 7% (*n* = 6) with 3, 7% (*n* = 6) with 4, and 3% (*n* = 3) with 5. Total satisfaction was thus evaluated by the sum of scale values 1 and 2, that is, 84% (Figure 4). 88% of patients would agree to further treatment and 92% would recommend the therapy to others. A selection of before and after photos proves the high satisfaction rates of our patient cohort. (Figures 5-7).

## DISCUSSION

This retrospective study at a single clinic with an FDA-cleared system (CoolSculpting Elite, Allergan Aesthetics AbbVie Company, Irvine, CA) for cryolipolysis therapy, confirms a high level of patient satisfaction and patient comfort. This study also documented cases of temporary side effects such as swelling, bruising, pain and a reduction in sensation which, however, within two weeks were completely regressive. The rapid healing corresponded to the results that were documented in the preceding studies.^12,13^ Furthermore, through this study a good level of patient comfort resulting from the pain level survey was reported. 78% of all patients rated the pain level as either no pain at all or light pain. The study showed that in 54% of all cycles abdomen and flanks represented the main body areas.

In our experience the establishment of a treatment plan and the choice of an optimal applicator are essential for desired treatment results, subsequent patient satisfaction and positive recommendation rate. Previous studies have already emphasised the importance of a treatment plan with the number of possible cycles for each individual body site and the treatment therefore.^14^

Compared to the patient survey data from Kilmer et al. we found nearly the same rate of patient satisfaction.^8^ While Kilmer et al. documented satisfaction of 83% of subjects, we found satisfaction in 84% of our cases (sum of scale values 1 and 2). While Kilmer et al. specifically investigated the submental region, our study revealed these promising results for various regions of the body. Inner and outer thigh studies showed that 93%, respectively 86% of subjects were satisfied.^15,16^

For the abdomen and flanks, Tan et al. found a patient satisfaction rate of 89.6%.^17^ This consistency of results proves the high overall satisfaction of this non-invasive procedure. Compared to Kilmer et al.’s findings we could document a slightly higher recommendation rate (92% in our cohort compared to 80% in the study of Kilmer et al.).

According to Kennedy et al., to date there are 5 FDA-approved non-invasive body contouring modalities: cryolipolysis, low-level laser therapy (LLLT), high-intensity focused electromagnetic field, radiofrequency (RF), and high-intensity focused ultrasound (HIFU).^18^ Although they do not achieve the same results as liposuction, they are an attractive alternative for patients who do not want the risks or costs associated with surgery.

Nevertheless, liposuction has to be mentioned as an invasive, alternative method of treatment. Compared to all above mentioned non-invasive therapies in the hand of an experienced board-certified plastic surgeon it is definitely a more powerful and a more precise tool.

Although we did not have a single case of paradoxical adipose hyperplasia (PAH) in our patient cohort, one must mention this possible side effect and deal with the patients’ associated concerns. Kilmer et al.’s investigation of the safety and efficacy of cryolipolysis of submental fat did not identify any study subjects with PAH, likewise.^8^ Nevertheless patients have to be advised, that this side effect does not occur with conventional liposuction. Incidence rates of PAH vary between 0.05% and 0.39%.^19^ In our experience, every patient still wanted to have the cryolipolysis treatment after having received an informed consent, especially with regard to PAH.

Since the lack of 3-dimensional volumetry represents a limitation of our study, we would suggest to further objectify the results of cryolipolysis therapy in upcoming studies. Besides the authors recommend the undertaking of further studies in order to better understand the underlying mechanisms of cryolipolysis.

## CONCLUSIONS

The results of this retrospective study from a single clinic show that cryolipolysis, carried out with the CoolSculpting Elite system is a safe and effective method for the treatment of subcutaneous fat deposits. During the study, no permanent adverse events occurred. Only temporary side effects were observed. With a suitable selection of patients and the establishment and implementation of an individual treatment plan, a high degree of patient satisfaction with a willingness to recommendation can be achieved.

## References

[1] International Society of Aesthetic Plastic Surgery (ISAPS), Global Survey, 2019. Accessed May 18, 2022. https://www.isaps.org/wp-content/uploads/2020/12/Global-Survey-2019.pdf

[2] American Society for Dermatologic Surgery (ASDS), Consumer Survey 2019. Accessed May 18, 2022. https://www.asds.net/Portals/0/PDF/consumer-survey-2019-infographic.pdf

[3] The Aesthetic Society’s Cosmetic Surgery National Data Bank: Statistics 2021. Aesthet Surg J. 2022;42 (Supplement_1):1-18. doi: 10.1093/asj/sjac116

[4] Duncan WC, Freeman RG, Heaton CL. Cold Panniculitis. Arch Dermatol. 1966;94:722-724. doi: 10.1001/archderm.1966.01600300046010 5923439

[5] Epstein EH, Jr, Oren ME. Popsicle Panniculitis. N Engl J Med. 1970;282:966-967. doi: 10.1056/NEJM197004232821709 5436034

[6] Rotman H. Cold Panniculitis in Children: Adiponecrosis E Frigore of Haxthausen. Arch Dermatol. 1966;94:720-721. doi: 10.1001/archderm.1966.01600300044009 5923438

[7] Manstein D, Laubach H, Watanabe K, Farinelli W, Zurakowski D, Anderson RR. Selective Cryolysis: A Novel Method of Non-invasive Fat Removal. Lasers Surg Med. 2008;40:595-604. doi: 10.1002/lsm.20719 18951424

[8] Kilmer SL, Burns AJ, Zelickson BD. Safety and Efficacy of Cryolipolysis for Non-invasive Reduction of Submental Fat. Lasers Surg Med. 2016;48(1):3-13. doi: 10.1002/lsm.22440 26607045 PMC5396277

[9] Ingargiola MJ, Motakef S, Chung MT, Vasconez HC, Sasaki GH. Cryolipolysis for Fat Reduction and Body Contouring: Safety and Efficacy of Current Treatment Paradigms. Plast Reconstr Surg. 2015;135(6):1581-1590. doi: 10.1097/PRS.0000000000001236 26017594 PMC4444424

[10] Krueger N, Mai SV, Luebberding S, Sadick NS. Cryolipolysis for Noninvasive Body Contouring: Clinical Efficacy and Patient Satisfaction. Clin Cosmet Investig Dermatol. 2014;7:201-205. doi: 10.2147/CCID.S44371 PMC4079633 25061326

[11] Avram MM, Harry RS. Cryolipolysis for Subcutaneous Fat Layer Reduction. Lasers Surg Med. 2009; 41( 10):703-708. doi: 10.1002/lsm.20864 20014262

[12] Stevens WG, Pietrzak LK, Spring MA. Broad Overview of a Clinical and Commercial Experience with CoolSculpting. Aesthet Surg J. 2013;33(6):835-846. doi: 10.1177/1090820X13494757 23858510

[13] Dierickx CC, Mazer JM, Sand M, Koenig S, Arigon V. Safety, tolerance, and patient satisfaction with noninvasive cryolipolysis. Dermatol Surg. 2013;39(8):1209-1216. doi: 10.1111/dsu.12238 23639062

[14] Altmann J, Burns AJ, Kilmer SL, et al. Global expert opinion on cryolipolysis treatment recommendations and considerations: a modified delphi study. Aesthet Surg J Open Forum. 2022;4:1-12. doi: 10.1093/asjof/ojac008 PMC9113840 35592181

[15] Few J, Saltz R, Beaty M, et al. Cryolipolysis: clinical best practices and other nonclinical considerations. Aesthet Surg J Open Forum. 2020;2(2):ojaa010. doi: 10.1093/asjof/ojaa010. doi: 10.1093/asjof/ojaa010. 33791637 PMC7671251

[16] Stevens WG, Bachelor EP. Cryolipolysis conformable surface applicator for non‐surgical fat reduction in lateral thighs. Aesthet Surg J. 2015;35(1):66-71. doi: 10.1093/asj/sju024 25568236 PMC4462597

[17] Zelickson BD, Burns AJ, Kilmer SL. Cryolipolysis for safe and effective inner thigh fat reduction. Lasers Surg Med. 2015;47(2):120-127. doi: 10.1002/lsm.22320 25586980 PMC6680208

[18] Tan T, Snell B, Braun M, et al. High participant satisfaction achieved using cryolipolysis for fat reduction of the abdomen and flanks. Aesthet Surg J. 2021;17:sjab421. doi: 10.1093/asj/sjab421 PMC9208824 34919631

[19] Kennedy J, Verne S, Griffith R, Falto-Aizpurua L, Nouri K. Non-invasive subcutaneous fat reduction: a review. J Eur Acad Dermatol Venereol. 2015;29(9):1679-1688. doi: 10.1111/jdv.12994 25664493

